# Outcome of faecal peritonitis in the ICU

**DOI:** 10.1186/cc11005

**Published:** 2012-03-20

**Authors:** J Sayer, G Simpson, L Mccrossan, I Welters

**Affiliations:** 1 Royal Liverpool University Hospital, Liverpool, UK

## Introduction

Faecal peritonitis often leads to intensive care admission. Anecdotally, patients with co-existing malignancy had an improved outcome. A retrospective analysis of all patients admitted to intensive care over 7 years was conducted to investigate this observation and identify factors that are associated with outcome from faecal peritonitis in intensive care.

## Methods

A retrospective analysis of all cases of faecal peritonitis admitted to the Royal Liverpool University Hospital ICU over 7 years. Clinical records, laboratory results, histology reports and radiological data were accessed. Statistical analysis was performed using chi-squared and Student's *t *tests.

## Results

A total of 133 patients were admitted to intensive care in 7 years. Thirty-six patients had underlying malignancy. Predicted mortality, indicated by APACHE II score, was similar in both groups (malignancy: 17.1, nonmalignancy: 16.2). Inpatient mortality was lower in patients with malignancy than those without (malignancy: 21.6%, nonmalignancy: 38.1%, *P *< 0.1) and shorter ITU stay (malignancy: 6.8 days, nonmalignancy: 12.7 days, *P *≤0.0005). Cancer patients required a shorter period of TPN or NG feeding (malignancy: 4.29 days, nonmalignancy: 7.7 days, *P *< 0.05), and a shorter duration of inotropic support (malignancy: 2.54 days, nonmalignancy: 4.44 days, *P *< 0.05). Peak inflammatory markers are lower in patients with malignancy, notably neutrophil count (malignancy: 21.15, nonmalignancy: 24.9, *P *< 0.05).

The mean APACHE II score was significantly lower in cases who survived, compared to those who did not (nondeaths: 15.3, deaths: 19.3, *P *< 0.005). Mean albumin at admission was similar for patients who survived compared to those who did not (deaths: 18.2, nondeaths: 18.6); however, minimum albumin during admission is significantly lower in patients who died than those who survived (deaths: 10.33, nondeaths: 13.24, *P *< 0.005). Duration of feeding support (TPN or NG feeding) and time to commencement of feeding showed no difference between patients who survived and those who did not.

## Conclusion

Underlying malignancy is associated with an increased survival, shorter ITU stay, less requirement for inotropic support and decreased inflammatory markers potentially due to a less aggressive inflammatory response as a consequence of the presence of malignancy. In this series, delay to introduction of nutrition and length of nutritional support are not associated with outcome; however, low albumin is associated with a poor outcome, although it is not clear if this is secondary to nutrition or inflammation.

